# Treatment of Ophthalmia, Neonatorum

**Published:** 1916-02

**Authors:** Lee Masten Francis

**Affiliations:** Buffalo. Associate in Ophthalmology, Med. Dept. Univ. of Buffalo. Attending Ophthalmologist, Buffalo General Hospital


### Treatment of Ophthalmia, Neonatorum

LEE MASTEN FRANCIS, M. D., F. A. C. S., Buffalo.

Associate in Ophthalmology, Med. Dept. Univ. of Buffalo.
Attending Ophthalmologist, Buffalo General Hospital.

It is neither within the intent or scope of this paper to discuss the treatment of ophthalmia neonatorum in all of its therapeutic or academic phases, but rather to put before you the principles underlying the handling of this disease, and to speak in a more or less general way of certain features which may concern you as general practitioners.

In the beginning, it seems wise to define ophthalmia of the new born. The term includes all cases of conjunctivitis, from what ever cause, occurring in infants during the first month of life. Mr. Sidney Stephenson of London in 1898, in a study based upon some 500 cases, adduced figures to the effect that while approximately 70% were due to the Neisser organism, the remaining 30% were due to other bacteria. The tables of other observers come somewhere near corroborating these proportions. In other words, contrary to the general impression of the medical world, nearly one-third of cases are non-gonorrhoel. In this one-third, one has to deal with either the staphlococci, streptococci, pneumococci, Koch,-Weeks—baccillus, the diplo-bacillus of Morax-Axenfied, Klebs-Loeffler, colon or some combination. The obstetrician who as a routine uses the Crede method, is also confronted with an occasional silver catarrh. This data is introduced for two reasons, first to remind us that there are ophthalmias other than gonorrhoel, a fact which has a practical bearing upon the reputation of physicians clientele, in that a conjunctivitis in a new born infant is not necessary indictment of the parents, and secondly to lead up to the importance of an early bacteriological diagnosis of the particular case in hand.

There are two classes of ophthalmia recognized by clinicians, based upon the time of infection; the primary in which infection takes place during birth, and the secondary, in which infection is received after delivery. In the former class, according to Weeks, gonorrhoel infection likely occurs during the passage of the child's head along the mother's tract, just at the time of delivery by the entrance of the vaginal secretion containing the gonococci into the conjunctival sac. In rare cases infection takes place ante-partum, the disease being well advanced at birth. While in the secondary class, that is those not infected during parturition, contamination by nurses or mothers hands, unclean washes, soiled linen and other infection carriers are responsible for a large number of these unfortunate cases. In the individual instance the time at which the disease makes its appearance may indicate whether the infection is vaginal or extra-vaginal, the period of incubation for gonorrhoel being from 12 to 48 hours.

**Diagnosis.**

While gonorrhoel cases have been known to appear within 12 hours, symptoms as a rule, do not present themselves until the second day and then in the form of a slight redness of the conjunctival. By the third morning the lids are glued together by mucos-pus. From then on the increase of pus, swelling of the lids, thickening of the conjunctival, and all of the other details of the well known picture of ophthalmia neonatorum present themselves. From what has been said before, it is quite obvious that bacteriological examination of the lid secretion is imperative. Just here it may be permitted to insist upon the importance of taking early smears. Those of us who have had experience in examining conjunctival secretions, will agree, that the best and most characteristic slides are obtained early in conjunctival disease. This is particularly true in gonorrhoea, as well as in some of the other organisms, which must be taken into a differential consideration. The platinum loup and slides should be as common a guest in an obstetrical bag as the hypodermic needle or chloroform bottle. The microscope and microscope alone, will determine in many instances the type of conjunctivitis with which we are dealing.

**Protection.**

It must be the first duty of the medical attendant when he discovers the presence of a one-sided conjunctival discharge to protect the second eye. This may be done by means of a shield of a watch crystal cemented on by adhesion tapes, reinforced by collodion, (an ordinary vacination shield affords a fair substitute), or by a cotton dam, extending from the forehead to the nose, likewise cemented by collodion. The child should be turned on the side of the infected eye to prevent secretions from trickling into the sound eye. The infants' hands and feet must be restrained to prevent carrying of infections by these means. The attendants must also be warned as to danger of contamination.

**Treatment.**

Treatment to be effective must be instituted early, the earlier the greater are the chances of success. Treatment resolves itself roughly into mechanical and germicidal. By the former is meant the removal of pus from the conjunctival sac. This is imperative. The conjunctival must be flushed with some germicidal solution, be it boric acid, sterile water, bichloride 1-10,000, normal saline, potassium permanganate, or what not. The choice of irrigation is of relatively slight significance, compared to the necessity of keeping the conjunctival free from secretion. These irrigations must be frequent and thorough enough to accomplish this end, ranging from every 15 minutes to every half hour or hour as the individual case may demand.

**Germicidal.**

Of almost secondary importance is the use of some germicial application other than the irrigation. Silver nitrate, in strengths from ½ to 4%, protargol, nargol, collargium, silver acetate, sophol, zinc chloride, alphozone, silver iodide and a host of others in varying strengths and in varying frequency, all have ardent and enthusiastic supporters. As a matter of fact the reader feels that silver nitrate is safe and well seasoned and with some of the newer silver salts as adjuncts, he is well armed. Where argyol* is not effective, some other is tried until the medicinent which does the best work in the individual case is found. These adjuncts may be used every hour by the attendant.

According to Axenfeld, the thermal range of the growth of the gonoccus is from 25 degrees to 39 degrees C (77 degrees to 102 degrees F). If this be true, cold such as may be safely applied to the eye cannot greatly inhibit the growth of that organism. Cold compresses, however, in the acute stage may relieve suffering. Cold should not be applied continually in the form of an ice bag, because of the danger of lowered corneal vitality but should be applied during alternate hours by means of gauze pledgets chilled on ice. Cold is absolutely contraindicated if the cornea becomes involved.

**Complications.**

The danger of and disastrous results from ophthalmia neonatorum are not in any way contingent upon damage to the conjunctiva, the primary host of the infection, but are the direct result of destruction of the cornea. Whether blindness or sight shall follow an attack, will depend entirely upon the preservation of the integrity of the cornea. While various complications may supervene, yet the success or failure of treatment must stand or fall upon the saving of a clear cornea. This is the really important factor to be considered and about the cornea the management of the case must revolve. Every other phase of the disease is of relative insignificance. The cornea must be watched with the greatest care and solicitude. The appearance of slight grayish haze or faintest abrasion is a danger flag of most vivid hue, for this likely is the fore runner of an ulcer, which in turn may mean an opaque scar or a lost eye. It is because of the cornea that

*Duane of New York has lately suggested that part of the efficiency of argyrol is due to its mechanical effect in removing infection. He therefore, advises the use of a drop of argyrol, then after waiting a few minutes, flushing the eye again removing the coagulated deep secretion. Finally another drop is instilled which is allowed to remain. In this way he feels that the secretions and bacteria are thoroughly removed and the argyrol that is last put in has unobstructed access to the germs that remain.

these cases should be under the care of the trained ophthalmologist. It is here that the most delicate and most intelligent management is most needed. In corneal involvement cold is absolutely contra indicated, while heat is most desired, along with many special measures ranging from atropine, and mild stimulants to paracentesis of the anterior chamber.

**Nursing.**

Just as important as medication, and in the minds of most of us of even more importance, is the necessity of careful nursing. Under no circumstances must the cornea be touched with either dropper, retractor or sponge. The reader is thoroughly convinced that many corneal ulcers are the direct result of abrasions of the corneal epithelium at the hands of careless nurses. When once the corneal epithelium is interrupted, an avenue of infection is created, producing an ulcer which may mean blindness to its unfortunate and guiltless host. The dry cotton which is sometimes used to wipe away secretion and take up the excess of irrigating fluid is a source of danger, in that the tiny cotton fibrils may become intangled between the lids and pressed against the cornea producing a dreaded abrasion. This may in large measure be obviated by insisting that cotton used for such purpose be wet. Thoughtful care and gentleness in nursing these cases aid beyond all estimate, while roughness and carelessness on the part of an attendant nullify the most intelligent medical efforts.

**General Treatment.**

While local measures are important, the reader is not so sure but that ophthalmologists as a class lose perspective in their anxiety about the eyes. Corneal resistance bears a direct relation to the vigor of the child. In an enthusiastic and commendable effort to control the local situation they momentarily forget that thy are treating a part of a body. There are undoubted instances where the frequent treatments which have seemed so necessary have so disturbed the little patient's rest that general health has suffered severely and along with the lowered vitality the cornea have melted away.

While this discussion has been in large measure one which has dealt with the gonorrheal type of opthalmia, it comes some where near being applicable in a general way to the other types of the disease, although most of them need less energetic measures; none except the diphtheretic and morax-axenfeld type need special mention. In the former anti-toxin is indicated. Unfortunately vacine therapy has brought no tangible asset in the treatment of gonorrhoel ophthalmia. In morax-axenfeld infection zinc in some form is an absolute specific.

The following summary may be made:

1. Not all opthalmias of the new born are gonorrheal Thirty per cent. are due to other organisms.

2. In cases of unilateral infection protect the sound eye.
3. Bacteriological examinations are most important. Smears should be taken early.
4. Early diagnosis is imperative. All cases of conjunctivitis in new born must be regarded with suspicion until proven of benign type.
5. Infection is not always during birth, but frequently comes from extra vaginal sources.
6. Because of the cornea, gonorrheal ophthalmia should be under the management of the ophthalmologist.
7. Careful and intelligent nursing is as important as medical advice.

It is generally conceded that successful treatment of ophthalmia neonatorum depends upon measures early instituted. For this reason great responsibility rests upon the general practitioner, not only for early recognition of the disease, but for insistance upon immediate advice and adequate nursing. In still a greater way may he be of infinite service to humanity by becoming a disseminator of information to his clientele and the public at large, concerning the dangers of ophthalmia neonatorium, and the fact that it is largely preventable by the Crede method. As far as the public is concerned this subject should no longer be handled with gloves but spoken of openly and frankly. By lay education as well as education of the medical public in intelligent prophylaxis may be stamped out the disease which is responsible for nearly a quarter of blindness.

575 Delaware Avenue.

---

**The Teaching of Hygiene and Maladies of First Infancy.** A. B. Marfan Le Nourrisson, Nov. 1914, pp 313-335. In the beginning of the twentieth century, of every 1,000 live-born infants in France, 150 died in the first year (of which 46 died in the first month, and 17 in the first five days), 50 in the second year, 25 in the third year, 17 in the fourth year, 13 in the fifth year; 56 in the fifth to tenth year, and 34 from the tenth to fifteenth year. The general mortality was 22 per 1,000, and of those under forty only 11 per 1,000 of the living died. In France at the beginning of the twentieth century about 800,000 infants were born every year, of which about 120,000 died before they were one year old; however, infant mortality has been on the decrease, as the following figures show:—

| | |
|---|---|
| From 1892-1895 | 258 per 1,000 |
| From 1896-1900 | 229 per 1,000 |
| From 1901-1905 | 164 per 1,000 |
| In 1906 | 145 per 1,000 |
| In 1909 | 143 per 1,000 |

